# Hospital Control and Efficiency

**Published:** 1905-03-11

**Authors:** 


### The Hospital.

A Journal of

The Medical Sciences and Hospital Administration.

Vol. XXXVII.—No. 963. MARCH 11, 1905.

### Hospital Control and Efficiency.

Dr. Mackintosh's paper, read before the Hospitals Association on the 3rd instant, should be carefully considered by the committees and officers of every voluntary hospital. Dr. Mackintosh made a very favourable impression upon his audience who felt that he had thoroughly mastered the subject and was entitled to be considered an authority upon the questions which he so ably dealt with. Dr. Mackintosh had the courage to go fully into details and to show the results which can be secured by attention to these matters on the part of the superintendent of a hospital. It is not enough to deal with the expenditure quarter by quarter, or month by month, or even week by week. To be effective the control must be exercised daily, and the superintendent must have the time, as well as the knowledge and will, to go into details thoroughly, and to put his finger at once upon the cause which may have produced an increase in expenditure in any part of the establishment. Where this is done systematically Dr. Mackintosh showed that the expenditure per bed and the expenditure in each department, as well as the cost of maintenance for the staff and the patients, can be so regulated as to be maintained year by year with practical uniformity. Wherever the accounts of a hospital exhibit great rises in expenditure in various directions, or continuous increases year by year, there it is practically certain that, in effect, no continuous or adequate control is exercised over the internal administration of that particular institution. It was said in the course of the discussion that everything which Dr. Mackintosh recommended was at present being done at the London and other hospitals. In the course of his reply, however, Dr. Mackintosh elicited the fact that in no London hospital is there a continuous daily control over expenditure. In the absence of this daily supervision of expenditure effective control is impossible, as it has undoubtedly proved to be, when tested by figures, at several London hospitals.

There was a general agreement that each hospital should be under the direct control of a superintendent. In the smaller hospitals, where the beds do not exceed two hundred and the burden of raising the necessary income cast upon the secretary is not too great, it was agreed that efficient control should be practically possible when vested in the hands of one officer—a secretary-superintendent. In the larger hospitals, however, the burden of raising income is often so great as to need the whole time and attention of an able secretary, who can command and should receive a high salary, for the work of the secretariat includes all the business of the hospital. If effective control is to be exercised over the internal affairs and expenditure of a great hospital, it follows that the appointment of a medical superintendent is essential. The discussion did not extend to the admission of patients and the discipline of the resident medical staff, though Dr. Mackintosh showed pretty effectively, that the presence of a medical superintendent was greatly helpful to the junior medical staff, highly beneficial to the patients, and promoted the smooth working and efficiency of the whole establishment. That Dr. Mackintosh's paper will do substantial service in the direction of economy and improvement of system in several hospitals is indicated by the circumstance that one result of the meeting was, we understand, to convince the representatives of one of the larger hospitals that the system of control and management which we have just explained is the best possible. We gathered, too, that there was a general feeling amongst those present that an officer of the experience and capacity of Dr. Mackintosh could not fail to give strength and efficiency to any large hospital fortunate enough to command his services, for they must result in such material economy that they could not fail to increase the efficiency and popularity of his institution. His system, as it has worked out at the Western Infirmary, Glasgow, proves, by the uniformity of the expenditure under all heads year by year, the effectiveness of the control exercised.

One interesting point revealed by the discussion was a failure to appreciate the difference in the conditions which prevail at the Royal Infirmary and the Western Infirmary, Glasgow. The latter institution, of which Dr. Mackintosh is medical superintendent, has been continuously improved and kept up to date in all departments. The Royal Infirmary is at present being worked under most unfavourable conditions, in relatively old buildings, the whole of which have been condemned, and are about to be demolished in order to clear the site for a new hospital, the plans of which are at present under discussion. In fact, then, the Royal Infirmary, Glasgow, may be regarded as being in a somewhat similar condition to that of the London Hospital before it was reconstructed and rebuilt. It follows that no useful or just comparisons can be made between the working and present condition of these two latter institutions, where the circumstances are necessarily wholly different, and where, in the case of the Royal Infirmary, matters are being carried on tentatively pending rebuilding, when no doubt the arrangements will be modernised and perfected in every department. There is always a danger in making comparisons between one hospital and another, unless all the circumstances are known and appreciated by those who make them. We shall never secure the adequate administration and control of our great hospitals with the maximum of efficiency, unless those engaged in investigations of this kind can be made to appreciate, at their proper value, all the material facts, in the absence of which any results arrived at must be valueless for practical purposes.

